# Characterising illness stages and recovery trajectories of eating disorders in young people via remote measurement technology (STORY): a multi-centre prospective cohort study protocol

**DOI:** 10.1186/s12888-024-05841-w

**Published:** 2024-05-30

**Authors:** Carina Kuehne, Matthew D. Phillips, Sarah Moody, Callum Bryson, Iain C. Campbell, Pauline Conde, Nicholas Cummins, Sylvane Desrivières, Judith Dineley, Richard Dobson, Daire Douglas, Amos Folarin, Lucy Gallop, Amelia Hemmings, Başak İnce, Luke Mason, Zulqarnain Rashid, Alice Bromell, Christopher Sims, Karina Allen, Chantal Bailie, Parveen Bains, Mike Basher, Francesca Battisti, Julian Baudinet, Katherine Bristow, Nicola Dawson, Lizzie Dodd, Victoria Frater, Robert Freudenthal, Beth Gripton, Carol Kan, Joel W. T. Khor, Nicus Kotze, Stuart Laverack, Lee Martin, Sarah Maxwell, Sarah McDonald, Delysia McKnight, Ruairidh McKay, Jessica Merrin, Mel Nash, Dasha Nicholls, Shirlie Palmer, Samantha Pearce, Catherine Roberts, Lucy Serpell, Emilia Severs, Mima Simic, Amelia Staton, Sian Westaway, Helen Sharpe, Ulrike Schmidt, EDIFY consortium, Heike Bartel, Tara French, Jonathan Kelly, Nadia Micali, Sneha Raman, Janet Treasure, Umairah Malik, Diego Rabelo-da-Ponte, Fiona Stephens, Tine Opitz, Nora Trompeter, Jessica Wilkins, Tamsin Parnell, Ruby Abbas, Alice Bromell, Grace Davis, Cameron Eadie, Lara Gracie, Beck Heslop, Katie McKenzie, Eniola Odubanjo, Chris Sims, Tallulah Street, Andreia Tavares-Semedo, Eleanor Wilkinson, Lucy Zocek

**Affiliations:** 1 Centre for Research in Eating and Weight Disorders, Institute of Psychiatry, King’s College London, Psychology & Neuroscience London (IoPPN), 103 Denmark Hill, First Floor, London, SE5 8AZ UK; 2 School of Health in Social Science, The University of Edinburgh, Edinburgh, UK; 3 Department of Biostatistics & Health Informatics, IoPPN, King’s College London, London, UK; 4 Social, Genetic & Developmental Psychiatry Centre, IoPPN, King’s College London, London, UK; 5 NIHR Maudsley Biomedical Research Centre, London, UK; 6 University College London, Institute of Health Informatics, London, UK; 7 Department of Forensic and Neurodevelopmental Science, IoPPN, King’s College London, London, UK; 8 EDIFY, London, UK; 9 South London and Maudsley NHS Foundation Trust, London, UK; 10 Cornwall Partnership NHS Foundation Trus, Bodmin, Cornwall UK; 11 Oxford Health NHS Foundation Trust, Oxford, Oxfordshire UK; 12 Cambridgeshire and Peterborough NHS Foundation Trust, Fulbourn, Cambridgeshire UK; 13 Bradford District Care NHS Foundation Trust, West Yorkshire, UK; 14 South West Yorkshire Partnership NHS Foundation Trust, Wakefield, UK; 15 Cumbria Northumberland Tyne and Wear NHS Foundation Trust, Newcastle Upon Tyne, UK; 16 Barnet, Enfield and Haringey Mental Health NHS Foundation Trust, London, UK; 17 Leeds and York Partnership NHS Foundation Trust, Leeds, UK; 18 Central and North West London NHS Foundation Trust, London, UK; 19 South West London & St. George’s Mental Health NHS Trust, St George’s Eating Disorders Service, London, UK; 20 Dorset Healthcare University NHS Foundation Trust, Poole, Dorset, UK; 21 Derbyshire Healthcare NHS Foundation Trust, Derby, Derbyshire, UK; 22 Norfolk and Suffolk NHS Foundation Trust, Norwich, Norfolk, UK; 23 Nottinghamshire Healthcare NHS Foundation Trust, Nottingham, UK; 24 North Staffordshire Combined Healthcare NHS Trust; Trentham, Staffordshire, UK; 25 NHS Lothian – NHS Scotland, Edinburgh, UK; 26 Devon Partnership NHS Foundation Trust, Exeter, Devon, UK; 27 Division of Psychiatry, Department of Brain Sciences, Imperial College London, London, UK; 28 Somerset Partnership NHS Foundation Trust, Taunton, UK; 29 Solent NHS Foundation Trust, Southampton, UK; 30 North East London NHS Foundation Trust, London, UK; 31 Division of Psychology and Language Sciences, University College London, London, UK; 32 Herefordshire and Worcestershire Health and Care NHS Trust, Worcester, UK

**Keywords:** Eating disorders, Recovery, Progression, Clinical staging, Remote measurement technology, Longitudinal monitoring, Prospective study, Observational cohort

## Abstract

**Background:**

Eating disorders (EDs) are serious, often chronic, conditions associated with pronounced morbidity, mortality, and dysfunction increasingly affecting young people worldwide. Illness progression, stages and recovery trajectories of EDs are still poorly characterised. The STORY study dynamically and longitudinally assesses young people with different EDs (restricting; bingeing/bulimic presentations) and illness durations (earlier; later stages) compared to healthy controls. Remote measurement technology (RMT) with active and passive sensing is used to advance understanding of the heterogeneity of earlier and more progressed clinical presentations and predictors of recovery or relapse.

**Methods:**

STORY follows 720 young people aged 16–25 with EDs and 120 healthy controls for 12 months. Online self-report questionnaires regularly assess ED symptoms, psychiatric comorbidities, quality of life, and socioeconomic environment. Additional ongoing monitoring using multi-parametric RMT via smartphones and wearable smart rings (‘Ōura ring’) unobtrusively measures individuals’ daily behaviour and physiology (e.g., Bluetooth connections, sleep, autonomic arousal). A subgroup of participants completes additional in-person cognitive and neuroimaging assessments at study-baseline and after 12 months.

**Discussion:**

By leveraging these large-scale longitudinal data from participants across ED diagnoses and illness durations, the STORY study seeks to elucidate potential biopsychosocial predictors of outcome, their interplay with developmental and socioemotional changes, and barriers and facilitators of recovery. STORY holds the promise of providing actionable findings that can be translated into clinical practice by informing the development of both early intervention and personalised treatment that is tailored to illness stage and individual circumstances, ultimately disrupting the long-term burden of EDs on individuals and their families.

**Supplementary Information:**

The online version contains supplementary material available at 10.1186/s12888-024-05841-w.

## Background

Eating disorders (EDs) are serious mental health conditions characterised by disturbances in eating behaviours, thoughts, and emotions, with significant physical and psychological consequences [1, 2]. Affecting about one in every six young females and one in 20 males, they pose a growing global public health concern, comparable to anxiety and depression [3, 4]. However, EDs have historically received little attention in research, leaving significant gaps in understanding their progression, variations in illness durations, and optimal treatment selection.

The peak onset of EDs occurs during the transitional period from adolescence to young adulthood, impacting socio-emotional, cognitive, and educational development [5]. This vulnerability is compounded by evidence suggesting that EDs are progressive disorders, where longer untreated illness duration is associated with poorer treatment outcomes [6, 7], greater symptom interconnectivity [8, 9], and neurobiological and behavioural changes that drive progression [10], altogether underscoring the critical importance of early intervention in ED management [11, 12].

Clinical staging models, that define the illness phenotypes along developmental lines with escalating symptom severity, offer a promising framework for understanding and intervening in the progressive nature of EDs [13, 14]. This contrasts with traditional approaches that view conditions as static and typically derive diagnostic criteria from advanced presentations, impeding early detection of the conditions in their nascent form. Establishing the underlying biopsychosocial processes at each stage that maintain illness, enhance progression or support recovery may inform stage-specific treatment to prevent further progression. These models, successfully adopted in psychiatry, including psychosis [15], are of current interest in EDs [16]. A proposed 4-stage model for Anorexia Nervosa (AN) ranges from an at-risk phase with attenuated symptoms to a chronic phase with severe, enduring symptoms [17]. However, variability remains in defining ED stages in terms of duration, symptom profiles, and treatment with much research solely focusing on AN.

The implication of staging models for prevention and early intervention proposes the possibility of symptom recovery at each stage, yet EDs exhibit low sustained recovery rates with only half achieving full remission with best available treatments [18]. This complexity is exacerbated by inconsistent conceptualisations of ED recovery that are predominantly biomedical (e.g., weight restoration, absence of ED behaviours), neglecting psychosocial dimensions and ED cognitions (e.g., subjective well-being, freedom from weight concerns) [19, 20]. Relapse risks persist until these underlying factors improve [21, 22]. Patients often describe their recovery as a protracted process with multiple ‘ups and downs’ that may take years to stabilise [23]. This intermediate state of partial improvement without regaining pre-illness health and functioning highlights the need for a more nuanced definition of ED recovery, using physical, behavioural, and psychological indices, and delineating partially and fully recovered groups.

Remote measurement technology (RMT) provides an unobtrusive, cost-efficient means to capture individuals’ daily behaviours and physiology using digital devices, gaining wider application in research across conditions [24, 25]. Active RMT enables delivery of smartphone-based assessments for detecting momentary changes. For instance, speech characteristics (incl. pitch, pauses, speaking rate) collected through smartphone microphones in app-based tasks can serve as scalable digital biomarkers of health outcomes, including depression severity, by providing information on cognitive, neuromuscular, and physiological aspects [26]. Passive RMT continuously gathers background data via smartphone and wearable sensors (e.g., location, heart rate, activity, screentime). The sensor data indicate behavioural markers relevant to clinical states (e.g., circadian rhythm, autonomic arousal, sociability). This range of domains measured by RMT reflects the proposed multidimensional nature of ED recovery, promising to elucidate the recovery process and outcome predictors. Research applying RMT to EDs is significantly lacking [27].

Through combining a traditional prospective cohort design with continuous remote monitoring, the STORY study (Illness Stages, Progression, and Recovery Trajectories of Eating Disorders in Young People) gathers comprehensive data from a large, deeply and dynamically phenotyped cohort of young people with a range of ED presentations. It will inform conceptual models of illness stages, progression, and recovery across illness durations, diagnoses, and age groups. STORY is part of the UKRI^1^-funded ‘EDIFY’ consortium which unites a UK-wide, multi-disciplinary team of investigators with the shared aim of improving prevention and early intervention for young people with EDs [28].

### Study objectives

The primary aim of the STORY study is twofold. The first (objectives 1–3) is to identify how biopsychosocial and neurocognitive symptom profiles differ between earlier and more progressed stages of EDs and which variables maintain illness, enhance progression or support recovery. The second (objectives 4–6) is to explore recovery processes and the factors that influence them by obtaining real-world data from participants’ daily lives.

- Objective 1: To use a multi-modal assessment protocol to cross-sectionally and longitudinally compare young people with earlier and later illness stages in terms of their biopsychosocial profiles and how these change over time within and across ED diagnostic groups.
- Objective 2: To identify baseline biopsychosocial predictors of outcome at 6 and 12 months within and across ED diagnostic and illness duration groups.
- Objective 3: To use cognitive tasks with illness-relevant stimuli to compare young people with earlier and later-stage illnesses in terms of their cognitive profiles over time within and across ED diagnostic and illness duration groups.
- Objective 4: To use biological and psychological RMT measures to compare young people presenting with an earlier-stage ED with healthy young people.
- Objective 5: To assess differences in recovery trajectories within and across ED groups.
- Objective 6: To identify early RMT predictors of ED recovery or lack of recovery at 12 months.

## Methods

### Study design

STORY is a multi-centre prospective cohort study, using ongoing remote monitoring for one year. Data will be collected via self-report online assessments at baseline, 6 and 12 months, via smartphones and wearable devices throughout the study period, and via neurocognitive measures completed in person by a subset of participants at baseline and 12 months. A further follow-up at 24 months is planned, recognising that ED recovery can continue over several years. These assessments are distinct from the main STORY study and not detailed in this protocol.

### Study sample

The total sample size target is 840 young people aged 16–25 years, capturing the critical period where EDs commonly manifest and progress while ensuring cognitive maturity to provide consent and complete study measures. Participants are divided into three groups based on symptom profiles and illness duration at baseline:

- ▶ 480 young people with an earlier-stage ED (illness duration ≤ 3 years); 
- ▶ 240 young people with a later-stage ED (illness duration > 3 years);
- ▶ 120 healthy controls (HCs).

The 3-year cut-off reflects more responsive treatment patterns in first-episode EDs of fewer than three years [29]. Symptom profiles distinguish between restricting-type presentations that involve severe limitations in food intake (e.g., Anorexia Nervosa [AN], Avoidant restrictive food intake disorder [ARFID]), and bingeing/bulimic-type presentations that involve episodes of binge eating, sometimes followed by compensatory actions, like purging or excessive exercise (e.g., Bulimia Nervosa [BN], Binge Eating Disorder [BED]). Individuals with atypical and subthreshold ED presentations (i.e., those exhibiting clinically significant symptoms without meeting full diagnostic criteria) are included to capture a comprehensive spectrum of ED symptomatology [30]. HCs have no current or past ED or other major mental disorders.

For the earlier-stage ED group, an estimated 100 recoveries within each of the two diagnostic groups are needed to test the predictive validity of RMTs, if 10 variables are to be entered into the predictive model [31]. Assuming a recovery rate of 50% at 12 months [18] and accounting for a 20% dropout rate, 480 participants are required to detect a medium-sized effect with 80% power (*f* = 0.15, α = 0.05; G*Power 3.1), aiming for an equal distribution of the two diagnostic groups.

The sample size for the later-stage ED group considers the total group for the comparisons between longer and shorter illness durations and varies between outcomes due to selective participation in some measures (e.g., neuroimaging). A sample of 480 provides 90% power to detect a small within-between group interaction effect (*f* = 0.08, *α* = 0.05), with two groups assessed twice (baseline, 12-months). Therefore, 240 participants with later-stage EDs will be recruited. A subsample of 100 for the additional in-person assessments, provides 95% power for small-medium interaction effects (*f* = 0.18, *α* = 0.05) with the two illness duration groups assessed at two time points.

A sample of 120 HCs represents 25% of the condition group for comparison analyses between control, ED and illness progression subgroups. The eligibility criteria are summarised in Table 1.

**Table 1:** Eligibility criteria for participation in STORY

Inclusion criteria	Exclusion criteria
*General inclusion criteria* Aged 16–25 Able to give informed consent for participation Willing and able to complete assessments via computer or smartphone Willing to use an Android smartphone as their only smartphone for the duration of the study *ED groups inclusion criteria* Meet DSM-5 diagnostic criteria for ED diagnosis (incl. AN, BN, BED, ARFID) or any other related eating or feeding disorder *HC group inclusion criteria* BMI > 18.5kg/m^2^ No current or past DSM-5 ED diagnosis No current or past major mental disorder (e.g., psychosis)	Major physical illness which impacts participants’ ability to participate in the study Insufficient knowledge of English to complete study assessments Severe learning disabilities Residing outside the UK Pregnancy

*DSM-5* The diagnostic and statistical manual of mental disorders, fifth edition, *BMI* Body mass index

### Study procedures

#### Recruitment

ED participants are recruited from an established network of 50 + FREED early intervention services^2^ and specialist child, adolescent and adult ED services across the UK. Clinicians conduct a preliminary assessment of the inclusion criteria and provide study materials to potential participants to review. Participants are also identified via primary care services, waiting lists of ED services, third-sector organisations (e.g., ED charities), schools and universities, relevant websites, social media, posters in public places, and existing research cohorts (e.g., ESTRA, GLAD and EDGI cohorts [32–34]). This wide recruitment strategy is hoped to allow for greater diversity in our sample than typically found in the research base, to ensure representation of various demographic groups, including those who do not commonly present to ED services (e.g., males, minoritised ethnic groups, those from the LGBTQ + community, those with higher body weight, those from rural locations) [35].

#### Screening

Interested individuals scan a QR code on recruitment materials linking to the online screening questionnaire to assess eligibility and inform group allocation (incl. sociodemographics, medical and ED history). Symptoms consistent with a current full or subthreshold diagnosis of an ED, as well as lack thereof for HCs, are confirmed via the Eating Disorder Diagnostic Scale (EDDS) [36]. ED illness duration is determined using adapted questions from the comprehensive onset interview used in FREED early intervention services [29]. Eligible participants are contacted by the research team and directed to an electronic consent form, where they can opt into optional study components. Researchers will follow up with participants where necessary, for instance, to confirm diagnoses, comorbidities or willingness to use the study devices.

#### Study assessments

Following consent, participants self-complete the online baseline assessments via Research Electronic Data Capture software (REDCap), a web application for managing online surveys [37]. These assessments are repeated at 6 and 12 months (see 2.4.1 for measures). REDCap sends automatic survey invitations and reminders to participants for the duration of the study.

At baseline, participants are sent an Android study smartphone (where not already owned) and Ōura smart ring (where consented) and attend an enrolment session online or in-person (subject to preference) with a researcher for assisted setup of the devices. The remote monitoring starts following the setup of the devices and lasts for 12 months (see 2.4.2 for active and passive measures).

**Fig. 1:**
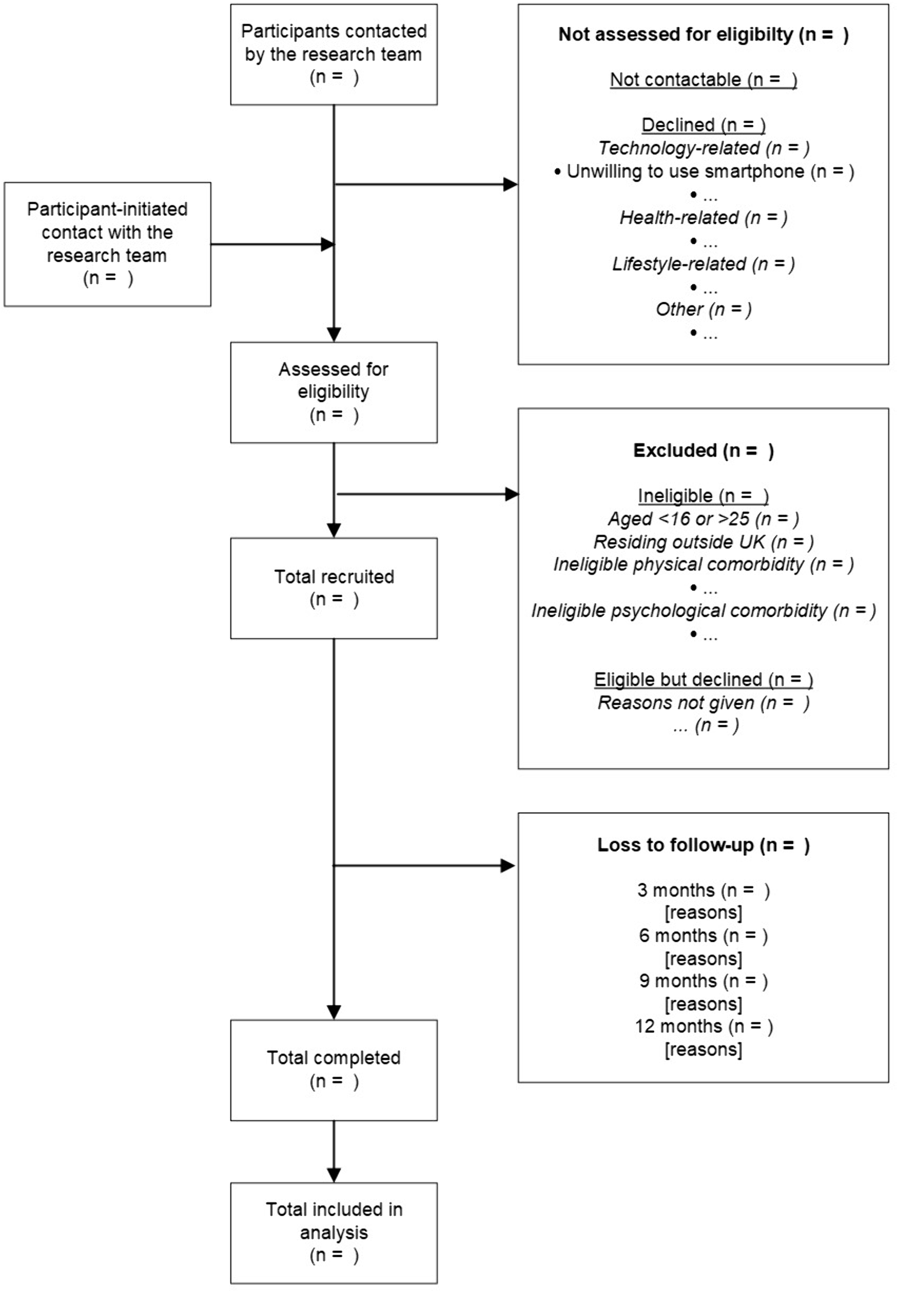
Participant flowchart

Additional optional in-person cognitive testing and neuroimaging assessments are completed at baseline and 12 months. Optional qualitative interviews are conducted at 6 and 12 months. See Table 2 for the complete schedule of observations and Fig. 1 for participant flow through the study.

**Table 2:** Schedule of events for STORY

Month	-1	0	1	2	3	4	5	6	7	8	9	10	11	12
**Pre-study procedures**														
Informed consent (R)	X													
Study enrolment session (V/I)		X												
**Screening assessments**														
Socio-demographics (R)	X							X						X
Medical history (R)	X													
ED history (R)	X													
ED diagnosis, EDDS (R)	X													
Medication and treatment (R)	X							X						X
**Remote data collection**														
Smartphone sensors (pRMT)			Continuous (month 1–12)											
Wearable sensors, Ōura ring^a^			Continuous (month 1–12)											
ED symptoms, ED-15 (aRMT)		X	XX	XX	XX	XX	XX	XX	XX	XX	XX	XX	XX	XX
Motivation to change, VAS (aRMT)		X	XX	XX	XX	XX	XX	XX	XX	XX	XX	XX	XX	X
Depression, PHQ-8 (aRMT)		X	XX	XX	XX	XX	XX	XX	XX	XX	XX	XX	XX	XX
Anxiety, GAD-7 (aRMT)		X	XX	XX	XX	XX	XX	XX	XX	XX	XX	XX	XX	XX
Speech (aRMT)		X	X	X	X	X	X	X	X	X	X	X	X	X
Weight (aRMT)		X	X	X	X	X	X	X	X	X	X	X	X	X
ESM assessment^b^ (aRMT)		X			X			X			X			X
**Outcome assessments**														
ED symptoms, EDE-Q (R)		X			X			X						X
Weight and appearance (R)		X												
Muscularity attitudes, DMS (R)		X						X						X
Psychological distress, PSYCHLOPS (R)		X						X						X
Mood profiles, POMS (R)		X						X						X
Affect, PANAS (R)		X						X						X
OCD symptoms, OCI-CV (R)		X						X						X
ASD traits, AQ-10 (R)		X												
Quality of life, WSAS-Y (R)		X						X						X
Loneliness, UCLA-4 (R)		X						X						X
Emotion regulation, DERS-16 (R)		X						X						X
Personality, SURPS (R)		X												
Mobile phone use (R)		X						X						X
Social media use, MSMU (R)		X						X						X
Alcohol use, AUDIT (R)		X						X						X
Smoking (R)		X						X						X
**Process evaluation**														
Qualitative interview^a^ (V)								X						X
**Additional neurocognitive outcomes** ^a^														
Attention to food stimuli (I)		X												X
Food choices (I)		X												X
Social attention (I)		X												X
Visual attention shifting (I)		X												X
Emotion matching (I)		X												X
Reward behaviour, PIT (I)		X												X
Inhibitory control, G/NG-T (I)		X												X
Resting state, ARSQ (MRI, ASL)		X												X
Reward-based learning, MID (MRI)		X												X
Impulse control, SST (MRI)		X												X
Brain states, movie-watching (MRI)		X												X

*(R)* REDCap web-based survey platform, *(V)* virtual via Microsoft Teams, *(I)* in-person, *(pRMT)* passive remote measurement app, *(aRMT)* active remote measurement app, *XX* delivered twice per month (every 2 weeks)

^a^optional; only completed by a subgroup of participants

^b^ *ESM* Experience sampling methodology; conducted every 12 weeks for 6 consecutive days, 6 times per day, *(MRI)* Task completed in scanner

### Remuneration

Participants receive a total of £50 for completing the online assessments (£20 at baseline, £15 for each follow-up), and £25 for completing the app-based assessments at the end of the data collection period. While the Ōura rings are to be returned by participants after the data collection periods, the study smartphone can be kept. In-person cognitive testing and neuroimaging assessments are reimbursed with £25 per assessment visit (adding up to an additional £100), plus travel costs. All monetary reimbursements are made via bank transfer.

### Ethical approval and consent to participate

STORY is conducted according to the Declaration of Helsinki and Good Clinical Practice, adhering to principles outlined in the NHS Research Governance Framework for Health and Social Care. Ethical approval was obtained in October 2023 from the London-Bloomsbury Research Ethics Committee (REC reference: 23/PR/0927). All staff working on the study have received training in study conduct, informed consent and risk assessment. All data is pseudonymised and stored securely in a research database per the General Data Protection Regulation.

Emphasis is placed on informed decision-making regarding participation and signed informed consent is obtained from all participants. Participants’ relationships with care teams are not impacted by participation or withdrawal from the study. If necessary, participants are signposted to third-sector organisations for additional support or encouraged to seek help in the NHS for clinical management.

### Outcome measures

Core outcome measures are grouped into online assessments measuring psychological, social, and functional outcomes at baseline, 6 and 12 months (see 2.4.1), and continuous active and passive RMT measures over the study period (see 2.4.2). Additional outcome measures include in-person cognitive and neuroimaging assessments at baseline and 12 months (see 2.4.3), and qualitative interviews at 6 and 12 months (see 2.4.4).

#### Online questionnaires

The primary outcome is the Eating Disorder Examination Questionnaire (EDE-Q) [38] global score at 12 months which provides data informative to the dual study aims of STORY investigating illness progression (higher scores indicating greater severity) and recovery (global score < 2.8; additional criterion of BMI > 18.5 kg/m^2^ for AN [39]).

Secondary outcomes are:

- ▶ ED-related attitudes and behaviours (Eating Disorder Scale, ED-15 [40]; six questions from the Avon Longitudinal Study of Parents and Children [41]).
- ▶ Motivation and readiness to change eating difficulties (two visual analogue scales; VAS).
- ▶ Muscularity-related attitudes (muscularity-oriented body image subscale of the Drive for Muscularity Scale, DMS [42]).
- ▶ Mood states and emotions (Profile of Mood States, POMS [43]; Positive and Negative Affect Scale, PANAS [44]).
- ▶ Depression symptoms (Patient Health Questionnaire; PHQ-8 [45]).
- ▶ Anxiety symptoms (Generalized Anxiety Disorder Questionnaire, GAD-7 [46]).
- ▶ Obsessive–compulsive symptoms (Obsessive Compulsive Inventory-Child Version, OCI-CV [47].
- ▶ Autistic traits (Autism Spectrum Quotient, AQ-10 [48]) at baseline only.
- ▶ Symptomatic and functional impairment (Psychological Outcome Profiles, PSYCHLOPS [49]; Work and Social Adjustment Scale–Youth-Version, WSAS-Y [50]).
- ▶ Emotion regulation difficulties (Difficulties in Emotion Regulation Scale, DERS-16 [51]).
- ▶ Loneliness (UCLA Loneliness Scale – Short form, UCLA-4 [52]).
- ▶ Addiction-reinforcing risk personality traits, e.g., impulsivity (Substance Use Risk Profile Scale, SURPS [53]) at baseline only.
- ▶ Mobile phone and social media use (13 questions from the Study of Cognition, Adolescents and Mobile Phones study [54], Motivations for Social Media Use Scale, MSMU [55]).
- ▶ Alcohol use (Alcohol Use Disorders Identification Test; AUDIT [56]) and smoking (two questions from Perman-Howe and colleagues [57]).

#### Remote data collection

Remote monitoring consists of active and passive components, following procedures established in previous research programmes [58, 59]. The open-source RADAR-base platform used to support the RMT data collection is described elsewhere [60].

##### Active RMT (aRMT) app

Participants install a purpose-built app that is part of the RADAR-base and was successfully applied in multiple projects. The app notifies participants to complete assessments according to the study schedule:

- ▶ ED symptoms and motivation to change eating difficulties every two weeks (ED-15 [40]; two VAS). Participants are invited to enter their weight monthly.

- ▶ Anxiety and depressive symptoms every two weeks (GAD-7 [46]; PHQ-8 [45]).
- ▶ Short speech tasks once a month, as used in previous studies [26]. A first scripted speech task asks participants to record themselves reading aloud excerpts from Aesop’s fable *“The North Wind and The Sun”* [61], which is reasonably phonetically balanced while relatively short, taking less than a minute to read aloud [62]. A second, free-response task asks participants to briefly speak about something they have coming up in the next week and how they feel about it (Appendix A). Participants can rerecord their response up to five times, if they are interrupted, or skip the task. The data is recorded, encrypted and uploaded to a secure server, then processed to extract linguistic and paralinguistic features (acoustic, prosodic, e.g., pitch, speaking rate, intensity) for analysis using similar pipelines to Cummins et al. and Zhang et al. [26, 63].
- ▶ Every 12 weeks, participants are prompted to complete brief in-the-moment assessments known as experience sampling method (ESM). ESM assesses mood changes, social interactions, and physical states in daily life. The schedule is initiated at six semi-random times per day within 90-min blocks between 08.30 and 22.00 for six consecutive days. Each ESM assessment consists of approximately 28 items and takes less than two minutes to complete (Appendix B). This intensity of assessment has demonstrated good acceptability in other clinical populations [64].

##### Passive RMT (pRMT) app

Participants install a second purpose-built pRMT app that is part of the RADAR base. This runs in the background and collects ongoing data via smartphone sensors, to test potential digital markers of change in ED symptoms and impairments. These include relative location data,^3^ ambient light and noise, weather conditions, sociability (e.g., via Bluetooth proximity data, length and duration of calls, keystrokes, number of text messages and emails), app use, and battery life. The pRMT app requires the Android operating system; participants who own non-compatible phones will be provided with Android smartphones.

##### Wearables sensors

Participants are invited to wear an ‘Ōura’ ring for the duration of the study (12 months), which collects ongoing data on sleep, autonomic arousal and physical activity, including heart rate, heart rate variability, step count, electrodermal activity, sleep efficiency, latency and fragmentation, skin temperature and oxygen saturation (SpO_2_). To access the Ōura app, participants enter deidentified login credentials generated by the research team. The Ōura app interface will not display any measured health data apart from the ring’s battery life and synch status. The pseudonymised data collected by the ring is synchronised with a smartphone app via Bluetooth, transmitted to Ōura Servers via WiFi, and then pulled to secure sFTP storage located in King’s College London.

The Ōura ring was selected due to the range of measurements available, improved accuracy in sleep tracking, competitive pricing, and ability to be safely implemented in an ED population (see 2.7). The Ōura ring has been shown to provide valid physical measurements comparable to gold-standard methods (e.g., polysomnography) in adult and adolescent populations [65, 66]. The minimal, aesthetically appealing design is aimed to minimise stigma and burden for the user.

#### Cognitive tasks and neuroimaging

*Reward behaviour*, *inhibitory control* and *food-related decision-making* are assessed via three cognitive tasks completed in person with a researcher present. These are a face-affective go/no-go task [67], a Pavlovian to Instrumental Transfer task [68], and a food choice task, where participants rate 42 food images for perceived healthiness and tastiness compared to a self-chosen ‘neutral’ reference item [69]. Additionally, participants complete the following five tasks utilising eye-tracking technology (Tobii TX300 eye tracker):

- ▶ Visual probe task [70]: Participants view high or low-calorie food items alongside resembling non-food objects, followed by a probe presented randomly over one stimulus which participants must respond to with a keypress. Response latency, time to first fixation and fixation duration are collected to assess *attentional biases* toward food cues.

- ▶ Two naturalistic scenes: Participants view a 124-s clip from the 1995-film ‘Welcome to the Dollhouse’ depicting a social situation of a young female attempting to find a table in a school cafeteria [71], followed by a 40-s clip of people being interviewed in the street [72]. During both videos, eye-tracking data will be collected to measure *social attention and comprehension*.
- ▶ Films Expressions Task [73]: Participants match a descriptive emotional verb (e.g., “shocked”) to a corresponding face image out of three, each being displayed for 500ms. Reaction times, accuracy and eye-movement data provide insight into participants’ emotion recognition abilities.
- ▶ Gap-Overlap task [74]: Participants view a centrally presented stimulus and then shift their attention to a peripheral stimulus presented randomly to either side. This task assesses the speed and accuracy of shifts of *low-level overt attention*. Attentional disengagement is manipulated via the timing and ordering of stimulus presentation, relative to a baseline condition.

Neuroimaging assessments include task-negative functional Magnetic Resonance Imaging (fMRI) and arterial spin labelling (ASL) to measure regional interactions in a resting state. The Amsterdam Resting-State Questionnaire (ARSQ) [75] is administered prior to the scan to measure *cognitive state and thought wandering*. Resting state scans also provide control images for the following tasks:

- ▶ Monetary Incentive Delay task [76]: Participants respond to visual stimuli to either win or avoid losing money, capturing neural substrates of different processing stages of *reward-based learning and motivation control* in the context of temporal discounting.
- ▶ Stop signal task [77]: Participants have to respond or withhold their response to a visual stimulus. The task yields an estimate of the participant’s reactive response inhibition serving as a proxy for *impulse control*.
- ▶ Movie-watching [78]: Participants watch a short clip from the movie ‘Despicable Me’ while in the scanner. This allows to measure *natural and real functional brain states* in response to continuous and immersive sensory stimulation that may not otherwise be detectable in traditional task-based designs.

#### Qualitative interviews

Participants are invited to online interviews at 6 and 12 months to investigate personal accounts of ED recovery. This information will complement quantitative data by offering a contextual understanding of individuals’ lived experiences and psychosocial dimensions of recovery (e.g., coping strategies). The interviews additionally serve as a process evaluation, exploring participants’ experiences within the study and RMT specifically. Understanding potential challenges and comfort levels with the study apps and devices will help refine and optimise their integration into future studies.

### Adverse events and study withdrawal

Due to STORY’s observational nature, it is not anticipated that participation increases significant risks of harm to participants. There may be several reasons for withdrawal from the study:

1. Participant chooses to no longer participate. Participants are informed of the voluntary nature of participation and their right to withdraw without providing a reason, with no impact on their care.
2. The research team withdraws the participant in the event of inter-current illness, adverse event, protocol violation, administrative or other reasons.
3. Participant loses capacity for continued participation.

Should a participant decide to withdraw from the study, efforts will be made to follow up to establish the reason for withdrawal to gather data on the acceptability of the study. Data from withdrawn participants will be included in the final analysis unless otherwise requested. In case of lack of engagement or missing data for more than three days, follow-up efforts will be made with participants via email and text message (if consented) up to three times before they are withdrawn from the study. Similarly, researchers will conduct random checks on the completion of aRMT measures, prompting participants as needed to ensure continued engagement and data quality.

### Statistical and analysis plan

The STORY study is exploratory and not using directional hypotheses. Analyses will be pre-registered (e.g., https://osf.io/) and any reports will clearly distinguish between a-priori and additional post-hoc/exploratory analyses. Datasets will be prepared, stored and shared in line with open science best practices and FAIR principles (www.go-fair.org/fair-principles) to allow replication.

To meet our first aim, various modelling approaches are used to characterise ED symptoms during illness progression and stages and identify outcome predictors. For example, network analysis methods are used that conceptualise factors (e.g., ED symptoms, comorbidities, other traits) as nodes and their associations as edges connecting the nodes to represent the psychopathology of EDs in a network of interconnected symptoms. To gain mechanistic insights and reveal differences that characterise ED subgroups and illness progression, our analyses further include comparisons between (i) controls, anorexic-type and bulimic-type subgroups, (ii) patient groups with different illness duration, and (iii) the initial and follow-up assessments.

To meet our second aim, features obtained from biosensors, questionnaires, tasks, and ESM assessments are used for analyses within and between groups. Initial raw data from smartphones and wearables is aggregated to generate feature sets. Time-independent and dependent probabilistic models are applied to investigate biological and psychological markers of recovery or illness progression, trajectory, and stage classification in EDs and identify predictors of outcome, including Mixture latent Markov (MLM) models. MLM models allow to identify unobserved subgroups (clusters) within the data that share similar symptom trajectories over time. This allows to explore how ED symptom patterns evolve differently across participant groups. Anomaly/novelty detection methods are used to investigate deviations from baseline data and the relationship between these changes and their symptoms.

Qualitative data is analysed using thematic analysis [79]. The thematic framework initially draws upon qualitative patient and public involvement (PPI) work conducted prior to STORY (see 2.7) and remains subject to development throughout analysis, as codes and themes are identified in the data.

The results of the study will be disseminated as widely as possible into the scientific and broader community, including via publications in peer-reviewed journals, scholarly book chapters, presentations at conferences, and publications in proceedings.

### Patient and public involvement

The original proposal of EDIFY was co-developed with eight young people with lived ED experience. The EDIFY project has a youth advisory board of 15 young experts-by-experience, six of whom are directly involved with the STORY study, having provided advice on STORY’s design (e.g., feasibility; attractiveness; questionnaire protocol; recovery definitions), and development of the study materials (e.g., designing documents; helping to avoid jargon; developing the recruitment video [https://www.youtube.com/watch?v=gRyVHnKYw4Y]). Youth advisors will continue to provide advice and feedback throughout the study.

Extensive pilot work has informed the acceptability of RMTs within STORY’s target population and its safe integration into the study. The perceived impact of RMT on weight- and food-related behaviours and attitudes was assessed as part of a qualitative interview study with former participants from the RADAR-MDD study who reported an ED diagnosis during their participation [58]. In an iterative process, the youth advisory board of the wider EDIFY consortium provided further in-depth feedback around the choice and integration of the wearable device. Overall, having access to measured health metrics was perceived to increase preoccupation with activity, weight and diet, thereby adversely impact ED symptomatology. In response to the feedback received, a smart ring was chosen as the wearable in STORY in contrast to other fitness-focused activity trackers used in similar studies (e.g., Fitbit, Garmin [31, 59]), and its use has been made optional. Additionally, access to data measured by the ring in the accompanying app can be restricted remotely by the research team allowing complete blinding.

## Discussion

While public and scientific awareness of EDs has grown over the past decades, the factors that perpetuate illness or are associated with sustained recovery remain poorly understood. STORY’s multidimensional data, capturing participants’ experiences in naturalistic everyday settings, will explore both neurobiological and psychosocial correlates of illness progression and recovery. This holds the potential for actionable results, paving the way for a more bespoke approach to treatment, aiming for earlier recovery and reduced chronicity. Integrating a qualitative component to complement the comprehensive quantitative assessments will foster a holistic understanding of recovery to shape interventions that resonate with individuals’ diverse needs.

The use of RMT in ED research is nascent and typically only over short periods [80–82]; its application to the STORY population and study duration is novel. The continuous monitoring of biopsychosocial factors promises to improve understanding of complex recovery processes and to explore under-researched factors potentially influencing ED progression, such as circadian rhythm and heart-rate variability [83, 84]. In the long run, these technologies could revolutionise clinical care. In contrast to existing ED treatment models, typically based on population effects or clinical expertise, personalised devices can monitor multidimensional outcomes and individual treatment responses in real time to inform clinical decisions (e.g., adjusting treatment type or intensity) [85]. Measurement-based care has proven effective in managing both physical and mental health conditions [86]. However, implementing RMT in an ED population presents unique challenges, most notably the use of wearable devices that are commonly associated with fitness and diet tracking. Such technology has been shown to trigger, maintain and worsen ED symptomatology in clinical and non-clinical populations [87, 88], mirrored in reluctance amongst individuals with an ED history to participate in RMT studies [89]. To understand how RMT can be safely integrated into ED research and clinical practice, we encourage future research to follow processes similar to those in STORY (e.g., PPI; close consultation with experts-by-experience; process evaluations).

The STORY study prioritises diverse representation by using liberal inclusion criteria and including groups commonly underrepresented in research, such as people of the global majority, individuals with under-researched EDs (e.g., ARFID) and those with persistent symptoms [35]. Individuals are eligible if they show significant ED symptoms at screening but have not been formally diagnosed yet which will help capture the full spectrum of ED experiences and severities. Recognising frequent psychological or neurodevelopmental comorbidities of EDs (e.g., mood or anxiety disorders, obsessive–compulsive disorder, autism), participants are not necessarily excluded for these unless significantly impaired or at safety risk. STORY further proactively explores diversity-related aspects (e.g., ethnicity, sexuality, gender, socio-economic background), to identify potential disparities in care and improve support for minority and marginalised groups. Finally, by encompassing an age range that straddles common divisions in research, policy, and service provision (i.e., < 18s vs. ≥ 18s), data from STORY allows for a more integrated and inclusive understanding of EDs in youth.

The STORY study is an ambitious project not without its challenges, primarily in participant recruitment and retention due to its longitudinal design, large number of variables measured, and transient study population. Recruitment challenges are likely to be eased by the wide reach of the study and broad inclusion criteria. To reduce attrition, participants are remunerated for individual assessments and allegiance to the study is fostered using purpose-designed study merchandise (e.g., tote bags, travel mugs), newsletters and events as successfully used in previous studies. Retention will be further aided by contact with dedicated research team members who provide technical support as needed, remind participants of the importance of data collection, and motivate them to contribute study data, as evidenced in previous longitudinal RMT studies [89]. The STORY study prioritises capturing young people’s experiences with EDs, enabling an in-depth exploration of individual factors related to illness progression and recovery. This focus excludes family or caregiver perspectives, known to influence ED development and recovery, and future research including both parties could provide valuable insights. However, focusing on individual experiences allows for a controlled design, avoiding potential biases introduced by family interactions during data collection.

Ultimately, the comprehensive data gathered from the STORY study, together with other initiatives within the EDIFY research programme, aspires to redefine the approach towards understanding and treating EDs. By spreading awareness and learning more about these disorders, we hope to identify them earlier and encourage people to seek help sooner, thereby fostering swifter recovery and diminishing long-term complications. Understanding the data-driven stories of young people with EDs is a crucial first step in rewriting those of young people in the future.

## Supplementary Information

Additional file 1. RMT Speech Task Instructions.

Additional file 2. Experience Sampling Methodology (ESM) assessment scheme.

## Data Availability

No datasets were generated or analysed during the current study.

## Abbreviations

AE: Adverse event
AN: Anorexia Nervosa
AQ-10: 10-item autism spectrum quotient questionnaire
ARFID: Avoidant Restrictive Food Intake Disorder
aRMT: Active remote measurement technology
ARSQ: Amsterdam Resting State Questionnaire
ASD: Autism spectrum disorder
ASL: Arterial spin labelling
AUDIT: Alcohol use disorders identification test
BED: Binge Eating Disorder
BMI: Body Mass Index
BN: Bulimia Nervosa
DERS-16: 16-item difficulties in emotion regulation scale
DMS: Drive for muscularity scale
DSM: Diagnostic and statistical manual
ED: Eating disorder
ED-15: Eating disorder scale
EDDS: Eating disorder diagnostic scale
EDE-Q: Eating disorder examination questionnaire
EDGI UK: Eating Disorders Genetics Initiative UK
EDIFY consortium: Eating Disorders: Delineating Illness and Recovery Trajectories to Inform Personalised Prevention and Early Intervention in Young People
ESM: Experience sampling method
ESTRA Study: Earlier detection and stratification of eating disorders and comorbid mental illnesses
fMRI: Functional magnetic resonance imaging
FREED: First episode and Rapid Early intervention for Eating Disorders
GAD-7: 7-item generalised anxiety disorder questionnaire
G/NG-T: Go/No-Go Task
GLAD Study: Genetic Links to Anxiety and Depression
GPS: Global positioning system
MID: Monetary incentive delay task
MLM: Mixture latent Markov model
MRI: Magnetic resonance imaging
MSMU: Motivations for social media use questionnaire
OCD: Obsessive compulsive disorder
OCI-CV: Obsessive Compulsive Inventory
PANAS: Positive and negative affect scale
PHQ-8: 8-item patient health questionnaire
PIT: Pavlovian to instrumental transfer task
POMS: Profile of mood states questionnaire
PPI: Patient and Public Involvement
pRMT: passive remote measurement technology
PSYCHLOPS: Psychological outcome profiles questionnaire
RADAR-CNS: Remote assessment of disease and relapse-Central nervous system
RADAR-MDD: Remote assessment of disease and relapse-Major depressive disorder
REC: Research Ethics Committee
REDCap: Research electronic data capture
RMT: Remote measurement technology
sFTP: Secure File Transfer Protocol
SST: Stop-signal task
SURPS: Substance use risk profile scale
UCLA-4: 4-item UCLA-Loneliness scale
VAS: Visual analogue scale
WSAS-Y: Work and social adjustment scale-Youth version

## Footnotes

1 UK Research and Innovation (UKRI) is a national funding agency investing in science and research in the UK.

2 First Episode Rapid Early Intervention for Eating Disorders (FREED).

3 GPS location data is obfuscated; that is, providing relative location data, not absolute coordinates, preventing identification of an individual’s home address or precise geographical location.
