# Supplementary material for: Characterising illness stages and recovery trajectories of eating disorders in young people via remote measurement technology (STORY): a multi-centre prospective cohort study protocol
Source: BMC Psychiatry. 2024 May 30;24:409. doi: 10.1186/s12888-024-05841-w (PMC11137943; doi:10.1186/s12888-024-05841-w)
Supplement: Supplementary file 1 — Additional file 1. RMT Speech Task Instructions. [file 12888_2024_5841_MOESM1_ESM.docx]

**Speech task protocol for STORY**

In the following two tasks, we would like you to record yourself speaking. To do this, first find a quiet place and make yourself comfortable.

You can sit or stand while you record yourself. Speak naturally in both tasks. Don’t try to change your voice or the speed at which you speak in any way.

1. Reading a short text

Below is a short excerpt from “*The North Wind and the Sun*”, one of Aesop’s fables. Press ‘START’, read out the text, then press ‘STOP’ when you have finished.

- The North Wind and the Sun were disputing which of them was stronger, when a traveller came along wrapped in a warm cloak. They agreed that the one who first succeeded in making the traveller take his cloak off should be considered stronger than the other. Then the North Wind blew as hard as he could, but the more he blew, the more closely did the traveller fold his cloak around him; and at last the North Wind gave up the attempt. Then the Sun shone out warmly, and immediately the traveller took off his cloak. And so the North Wind was obliged to confess that the Sun was the stronger of the two.

***Source:*** *International Phonetic Association, Handbook of the International Phonetic Association: A guide to the use of the International Phonetic Alphabet. Cambridge University Press, 1999.*

1. Speaking freely^[[1]](#footnote-2)^

Next, we would like you to record yourself answering a question. You can answer the question however you want. This recording cannot be used to send any kind of message to the STORY team. If you need help, please contact the research team directly ([story@kcl.ac.uk](mailto:story@kcl.ac.uk)).

Please talk about something you have coming up next week, how you feel about it and why.

- What is it that you have coming up? An event, an appointment, a holiday, a meeting, a celebration, a deadline, …
- How do you feel about this event or activity, and why?

You can speak for up to 90 seconds.

Press ‘START’, read out the text, then press ‘STOP’ when you have finished.

**RADAR Active App: Speech task screenshots**


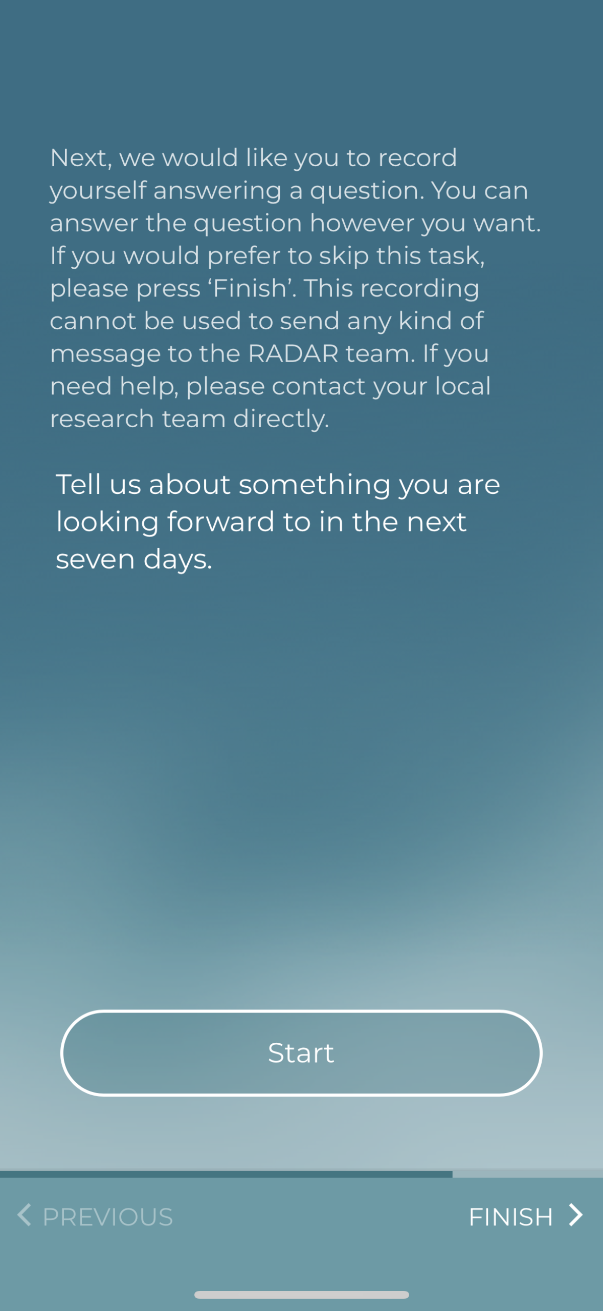


Speaking freely


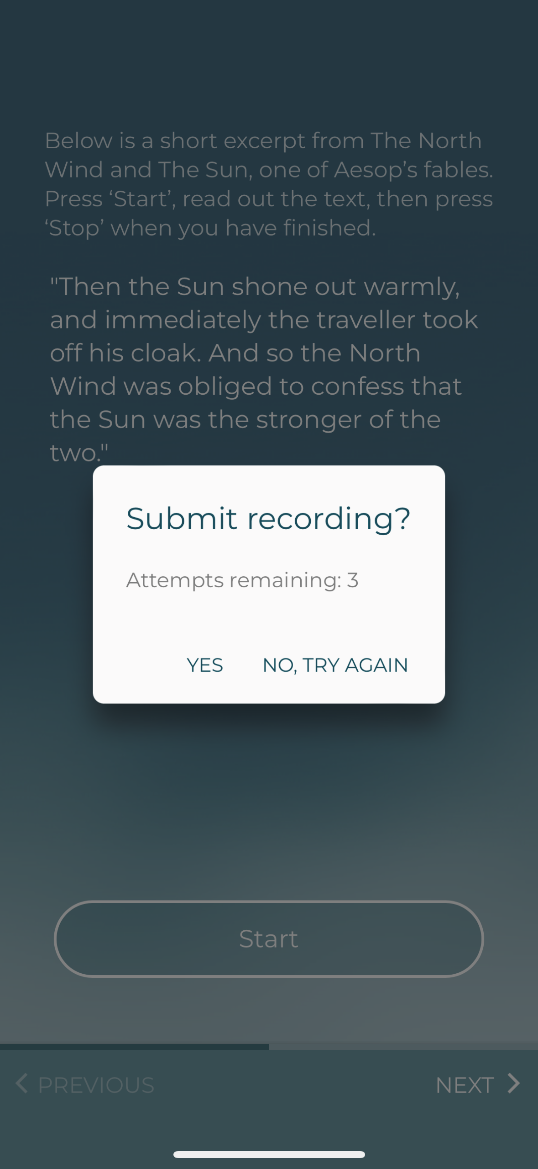


Submit recording

4 attempts in total
(4^th^ will be submitted)


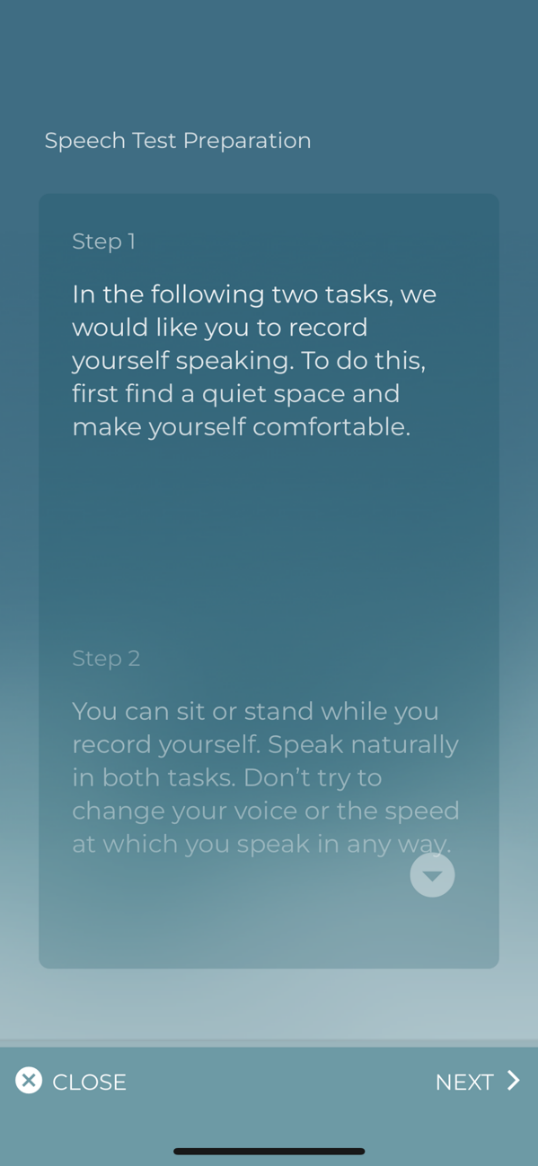


Task description


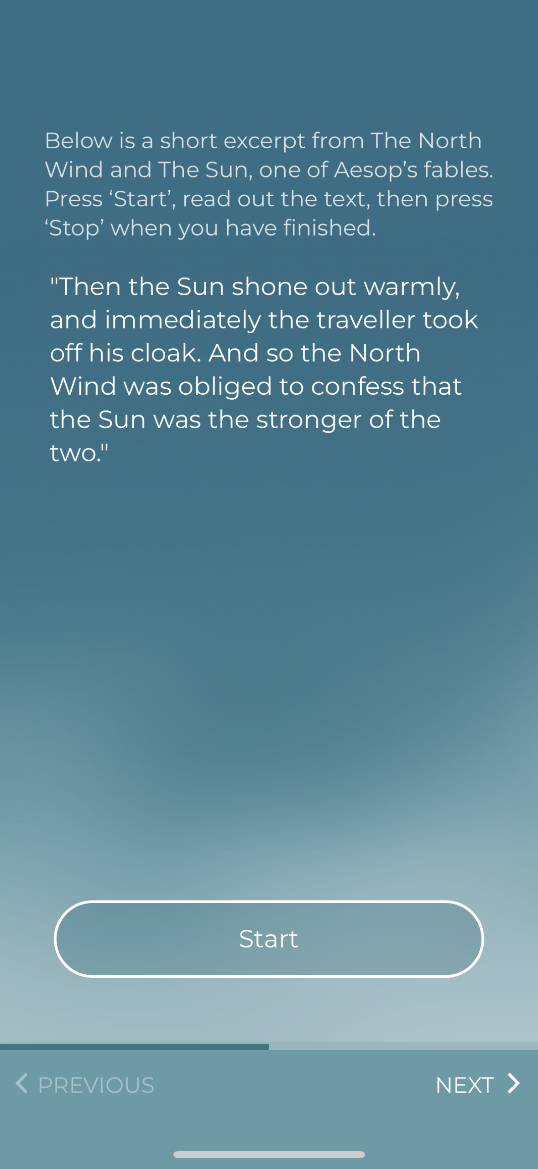


Instructions (2)

Reading a short text

Overview of the speech timing measures to be used in the analysis.

| **Feature** | **Definition** |
| --- | --- |
| Recording Duration | Total length of file; i.e., pauses/silence and spoken content |
| Phonation Time | Length of spoken content within the file |
| Phonation Ratio | Phonation Time divided by Recording Duration |
| Number of Syllables | Number of syllables automatically identified within the file |
| Number of Pauses | Number of pauses automatically identified within the file |
| Speaking Rate | Number of Syllables divided by Recording Duration |
| Articulation Rate | Number of Syllables divided by Phonation Time |
| Mean Length Run | Number of Syllables divided by the Number of Pauses |
| Pause Rate | Number of Pauses divided by Recording Duration |
| Average Syllable Duration | Phonation Time divided by the Number of Syllables |
| Average Pause Duration | Length of all pauses in file divided by the Number of Pauses |

Overview of the prosodic and phonation features to be used in the analysis (extracted using default Praat Settings).

| **Feature** | **Definition** |
| --- | --- |
| Pitch (Mean) | The auditory perception of tone within a speech signal. Extracted using Praat’s ’Sound: To Pitch (ac)’ function |
| Pitch (Std. Dev.) |  |
| Intensity (Mean) | Loudness of speech signal (measured in dB)  Extracted using Praat’s ’Sound: To Intensity (ac)’ function |
| Harmonic to Noise Ratio (Mean) | The extent to which noise affects the harmonic structures in a voice signal  Extracted using Praat’s ’Sound: To Harmonicity (cc)’ function |
| Jitter (Mean) | Variations in signal frequency caused by irregular vocal fold vibration.  Extracted using Praat’s ’Get jitter (local)’ function |
| Shimmer (Mean) | Variations in signal frequency and amplitude caused by irregular vocal fold vibration.  Extracted using Praat’s ’Get shimmer (local)’ function |
| Fraction Unvoiced Frames | Fraction of speech frames produced where the vocal folds are not vibrating  Extracted from Praat’s ’Voice Report’ |
| Number of Voice Breaks | Sudden abnormal shifts in pitch in a voice signal  Extracted from Praat’s ’Voice Report’ |
| Degree of Voice Breaks | The degree of these abnormal shifts  Extracted from Praat’s ’Voice Report’ |

**Speech task pilot and PPI work**

Youth advisor feedback

- 60-90 seconds is a good length, it’s long enough to cover all the aspects of the question but limited enough to not feel overwhelmed or get sidetracked.
- Instructions cause initial feelings of uncertainty / scepticism, but once started, the words started flowing and filling 90 seconds was easy. Felt in fact good to get feelings off their chest.
- The prompt “How does this make you feel” followed by “Why do you feel that way” is useful for self-reflection and can help to 'untangle' any negative feelings about the described event.
- Talking about an event and admitting out loud the feelings caused by it can cause feelings of vulnerability, even when the event itself is not emotional. It was mentioned that this sense of vulnerability could possibly make it hard for some participants to find the words and cause skipping the task entirely.

1. Participants will complete this speech task for the first time together with a researcher during their initial onboarding session. A researcher will demonstrate how to complete this task using a fictional character talking about a fictional event (*example:* Batman talking about planning to save the citizens of Gotham from a villain, and feeling proud about this because it gives his life a purpose and meaning). [↑](#footnote-ref-2)
