# Supplementary material for: Characterising illness stages and recovery trajectories of eating disorders in young people via remote measurement technology (STORY): a multi-centre prospective cohort study protocol
Source: BMC Psychiatry. 2024 May 30;24:409. doi: 10.1186/s12888-024-05841-w (PMC11137943; doi:10.1186/s12888-024-05841-w)
Supplement: Supplementary file 2 — Additional file 2. Experience Sampling Methodology (ESM) assessment scheme. [file 12888_2024_5841_MOESM2_ESM.docx]

**Experience sampling methods protocol for STORY**

| Question | Scale option |
| --- | --- |
| 1. I slept well. *[once per day]* | Not at all 0 1 2 3 4 5 6 7 Very much |
| 1. Right now, I feel cheerful. | Not at all 0 1 2 3 4 5 6 7 Very much |
| 1. Right now, I feel down. | Not at all 0 1 2 3 4 5 6 7 Very much |
| 1. Right now, I feel anxious. | Not at all 0 1 2 3 4 5 6 7 Very much |
| 1. Right now, I feel relaxed. | Not at all 0 1 2 3 4 5 6 7 Very much |
| 1. Right now, I feel irritated. | Not at all 0 1 2 3 4 5 6 7 Very much |
| 1. Right now, I feel stressed. | Not at all 0 1 2 3 4 5 6 7 Very much |
| 1. Right now, I feel content. | Not at all 0 1 2 3 4 5 6 7 Very much |
| 1. Right now, I feel insecure. | Not at all 0 1 2 3 4 5 6 7 Very much |
| 1. Right now, I feel hopeful. | Not at all 0 1 2 3 4 5 6 7 Very much |
| 1. Right now, I feel lonely. | Not at all 0 1 2 3 4 5 6 7 Very much |
| 1. Right now, I feel hungry. | Not at all 0 1 2 3 4 5 6 7 Very much |
| 1. Right now, I feel full. | Not at all 0 1 2 3 4 5 6 7 Very much |
| 1. Right now, I am satisfied with how I look. | Not at all 0 1 2 3 4 5 6 7 Very much |
| 1. Right now, I feel the urge to restrict. | Not at all 0 1 2 3 4 5 6 7 Very much |
| 1. Right now, I feel the urge to purge. | Not at all 0 1 2 3 4 5 6 7 Very much |
| 1. Right now, I feel the urge to overeat. | Not at all 0 1 2 3 4 5 6 7 Very much |
| 1. Since the last beep, I have fasted or restricted what I’ve eaten in order to control my weight. | Not at all 0 1 2 3 4 5 6 7 Very much |
| 1. Since the last beep, I have binged.   *(I felt out of control of my eating and ate an unusually large amount of food given the circumstances)* | Not at all 0 1 2 3 4 5 6 7 Very much |
| 1. Since the last beep, I have made myself sick (vomit) or used laxatives as a means of controlling my weight. | Not at all 0 1 2 3 4 5 6 7 Very much |
| 1. Since the last beep, I have exercised excessively in order to control my weight. | Not at all 0 1 2 3 4 5 6 7 Very much |
| 1. Right now, I feel restless. | Not at all 0 1 2 3 4 5 6 7 Very much |
| 1. Physically, I am tired. | Not at all 0 1 2 3 4 5 6 7 Very much |
| 1. I am able to concentrate well. | Not at all 0 1 2 3 4 5 6 7 Very much |

| 1. What am I doing (just before the beep)? | - Nothing; - Resting; - Working / studying; - Passive leisure (TV, computer, videogames, on social media / apps, reading); - Exercising / sports; - Eating / drinking; - Travel / transport; - Household chores / groceries; - Self-care; - Interacting with people face-to-face; - Interacting with people online (texting, calling or video calling, on social media); - Something else *(please describe)* |
| --- | --- |
| 1. Where am I? | - At home; - At family or friends’ place; - At work, school, university or college; - Travelling somewhere / in a vehicle; - In a healthcare facility; - Somewhere else indoors *(please describe)*; - Somewhere else outdoors *(please describe)* |
| 1. Who am I with? | - Alone; - Partner; - Relatives living with you; - Relatives not living with you; - House / roommates; - Friends; - Classmates / colleagues; - Caregiver; - Strangers / others; |
| 27_a1. How many people am I with? | 1; 2 ; 3-10; over 10 |
| 27_a2. I am comparing how I look with the others around me right now. | Not at all 0 1 2 3 4 5 6 7 Very much |
| 27_a3. I would prefer to be alone. | Not at all 0 1 2 3 4 5 6 7 Very much |
| 27_a4. I feel connected to the people I am with. | Not at all 0 1 2 3 4 5 6 7 Very much |
| 27_b1. I feel fine being alone. | Not at all 0 1 2 3 4 5 6 7 Very much |
| 27_b2. I would prefer to be with others. | Not at all 0 1 2 3 4 5 6 7 Very much |
| 27_b3. Being alone right now is my choice. | Not at all 0 1 2 3 4 5 6 7 Very much |
| 1. I feel left out. | Not at all 0 1 2 3 4 5 6 7 Very much |

Text in colour represents display logic:

- Orange text [items 27_a1 – 27_a4] will only be displayed if the first orange answer in question 27, ‘alone’, is selected.
- Blue text [items 27_b1 – 27_b3] will only be displayed if any of the blue answers in question 27, ‘partner’ ‘relatives living with you’ ‘relatives not living with you’ house/roommates’ ‘friends’ ‘classmates/colleagues’ ‘caregiver’ ‘strangers/others’, are selected.
